# Structural and functional plasticity specific to musical training with wind instruments

**DOI:** 10.3389/fnhum.2015.00597

**Published:** 2015-10-29

**Authors:** Uk-Su Choi, Yul-Wan Sung, Sujin Hong, Jun-Young Chung, Seiji Ogawa

**Affiliations:** ^1^Neuroscience Research Institute, Gachon University of Medicine and ScienceIncheon, South Korea; ^2^Kansei Fukushi Research Institute, Tohoku Fukushi UniversitySendai, Japan; ^3^Reid School of Music, Edinburgh College of Art, Institute for Music and Human Society Development, University of EdinburghEdinburgh, UK

**Keywords:** cortical thickness, resting-state network, wind instruments, musical training, neuronal plasticity

## Abstract

Numerous neuroimaging studies have shown structural and functional changes resulting from musical training. Among these studies, changes in primary sensory areas are mostly related to motor functions. In this study, we looked for some similar functional and structural changes in other functional modalities, such as somatosensory function, by examining the effects of musical training with wind instruments. We found significant changes in two aspects of neuroplasticity, cortical thickness, and resting-state neuronal networks. A group of subjects with several years of continuous musical training and who are currently playing in university wind ensembles showed differences in cortical thickness in lip- and tongue-related brain areas vs. non-music playing subjects. Cortical thickness in lip-related brain areas was significantly thicker and that in tongue-related areas was significantly thinner in the music playing group compared with that in the non-music playing group. Association analysis of lip-related areas in the music playing group showed that the increase in cortical thickness was caused by musical training. In addition, seed-based correlation analysis showed differential activation in the precentral gyrus and supplementary motor areas (SMA) between the music and non-music playing groups. These results suggest that high-intensity training with specific musical instruments could induce structural changes in related anatomical areas and could also generate a new functional neuronal network in the brain.

## Introduction

Structural and functional changes in the human brain resulting from musical training have been noninvasively studied using various imaging modalities, including magnetic resonance imaging (MRI; Schlaug, 2001; Münte et al., 2002; Schmithorst and Wilke, 2002; Schlaug et al., 2005, 2009; Hyde et al., 2009; Penhune, 2011; Steele et al., 2013). Two main approaches have been used in MRI studies: structural and functional. For structural approaches, diffusion tensor imaging (DTI), cortical thickness analysis, and voxel-based morphometry (VBM) have been widely used.

Changes in the white matter (WM) of musicians have been demonstrated by DTI (Schlaug et al., 1995; Rüber et al., 2015). Musicians often show different fractional anisotropy (FA) maps in several brain areas compared with non-musicians (Schmithorst and Wilke, 2002; Bengtsson et al., 2005; Halwani et al., 2011; Engel et al., 2014; Moore et al., 2014). For example, they showed lower FA maps compared with non-musicians in the corticospinal tract, corona radiate, and internal capsules bilaterally (Schmithorst and Wilke, 2002; Imfeld et al., 2009), but higher FA maps in the right posterior limb of the internal capsule (Han et al., 2009) and corpus callosum (Schmithorst and Wilke, 2002; Steele et al., 2013). FA values are often used to study the strength of the WM fiber tract. Low FA values mean weak connectivity of the fiber tract, and high FA values mean strong connectivity of the fiber tract (Schmithorst and Wilke, 2002). Even within some groups of musicians, different FA maps can be found depending on whether or not they have absolute pitch (AP), which is the ability to detect a pitch without any tonal reference (Oechslin et al., 2010). Musicians with AP showed significantly higher FA maps compared with those without it in the subgyral WM of the right temporal lobe, particularly within the path of the inferior fronto-occipital and inferior longitudinal fasciculus (Dohn et al., 2015).

Changes in the gray matter (GM) have been demonstrated by cortical thickness analysis and VBM. It has been reported that musicians have greater cortical thickness in the superior temporal and dorsolateral frontal regions as a result of their training (Bermudez et al., 2009). Musicians who were trained at a younger age showed greater cortical thickness in the ventral premotor cortex compared with those who were trained at an older age (Bailey et al., 2014). Among the other brain areas that showed different cortical thickness, Heschl's and bilateral intraparietal sulci strongly correlated with musical performances in relative pitch tasks (Foster and Zatorre, 2010). Along with cortical thickness analysis, VBM is also a very useful tool for finding structural changes. Musicians have been shown to have a significant increase in the volume of GM in the right fusiform (James et al., 2014), right posterior and middle cingulate, right and left superior temporal (Schneider et al., 2002; Bermudez and Zatorre, 2005), and right inferior orbitofrontal gyri as well as in the Broca's area in the left inferior frontal gyrus (IFG) that is associated with music-related abilities (Sluming et al., 2002; Fauvel et al., 2014), premotor cortex (Gaser and Schlaug, 2003), cerebellum (Hutchinson et al., 2003), and hippocampus (Groussard et al., 2010). Musicians who were trained before the age of seven showed an increased volume of the vPMC compared with musicians who were trained at an older age (Bailey et al., 2014).

For functional studies, activation of brain areas and functional connectivity among them have been measured to examine neuronal differences between the musicians and non-musicians. A representative functional difference between the musicians and non-musicians in functional MRI (fMRI) studies showed strong activation in auditory and motor areas, which are known to play crucial roles in musical activities (Baumann et al., 2005). Other studies have reported that non-musicians show a significant decrease in the brain activation in the right superior temporal gyrus when listening to trained melodies (Chen et al., 2012). Among musicians, enhancement of functional activity was found in the left middle and superior temporal gyri, left IFG, and right ventromedial prefrontal cortex in response to pattern deviation (Habermeyer et al., 2009). While performing musical tasks, musicians showed increased audiovisual asynchrony responses and effective connectivities in their superior temporal sulcus-premotor-cerebellar circuitry (Lee and Noppeney, 2011). Musicians also showed different functional connectivities not only during the task but also at rest. Furthermore, musicians also showed strong functional connectivity among the cingulate, right prefrontal cortex, and left temporal pole (Fauvel et al., 2014).

Most previous studies investigating either structural or functional changes in the brain have focused on musicians who play keyboard (Jäncke et al., 2000; Krings et al., 2000; Itoh et al., 2001; Haslinger et al., 2004; Meister et al., 2004; Parsons et al., 2005; Bengtsson et al., 2005; Baumann et al., 2007; Han et al., 2009; Gärtner et al., 2013; Engel et al., 2014; Alves-Pinto et al., 2015) or string instruments (Kim et al., 2004; Norton et al., 2005; Bangert and Schlaug, 2006; Vaalto et al., 2013; Verrel et al., 2013; Vollmann et al., 2014; Rüber et al., 2015). The instruments in those studies require the use of hands, and brain areas that showed specific functional and structural changes in those studies were related to motor activities. These functional studies reported different brain activation associated with motor practice (Jäncke et al., 2000; Haslinger et al., 2004) and functional connectivity between auditory and motor areas (Krings et al., 2000). Structural changes were also shown in both WM (Bengtsson et al., 2005) and GM (Han et al., 2009).

While many previous music-related studies have been conducted, none has examined neuroplasticity relating to wind instruments, which mainly require the mouth (lips and tongue) to play, in contrast to instruments described above, which mainly use other body parts, such as hands. Although the mouth is also used for singing (Sundberg, 1975; Ladefoged, 1978; Sundberg and Rossing, 1990), mouth usage while singing is different from playing wind instruments because the mouth interacts with objects when playing the latter (Gallivan and Eitnier, 2006; Wolfe et al., 2009).

In the current study, we hypothesized that similar structural and functional changes would occur in other brain modalities, such as the somatosensory, and examined the effects of musical training with the subjects who play wind instruments (“music playing group”) compared with the subjects who do not play music (“non-music playing group”). Wind instrument players are expected to develop their lips and tongue touches. Therefore, we assumed that the structures of somatosensory areas of the brain that are associated with the lips and tongue would differ between the music and non-music playing groups. In addition, we performed a resting-state fMRI analysis to investigate the possibility of functional connectivity modifications due to musical training.

## Materials and methods

### Participants

Fourteen music playing [all female; mean age ± standard deviation (SD), 20.35 ± 1.21 years] and 14 non-music playing subjects (all female; mean age ± SD, 20.14 ± 1.23 years) participated in this study. Those included in the music playing group played wind instruments (woodwinds and brass) for more than 7 years (mean ± SD, 7.93 ± 1.21 years) as members of junior and senior high school and university wind ensembles. In contrast, non-music playing subjects did not have any extra musical experience except for regular music classes in school.

None of the subjects had a history of neurological disease or any medical conditions (i.e., pregnancy, use of a cardiac pacemaker, or claustrophobia). After subjects were given a complete description of the study, written informed consent was obtained in accordance with the Declaration of Helsinki. This study was approved by the Institutional Review Board of Tohoku Fukushi University (Japan).

### MRI data acquisition

All subjects were scanned in two sessions that included structural (T1) and functional imaging (resting-state fMRI). Structural images were acquired using the following parameters: repetition time = 1900 ms, echo time = 2.52 ms, matrix size = 256 × 256, in-plane resolution = 1 × 1 mm^2^, slice thickness = 1 mm, and number of slices = 192. Resting-state fMRI data were acquired using the following parameters: repetition time = 2000 ms, echo time = 30 ms, matrix size = 64 × 64, in-plane resolution = 3.4 × 3.4 mm^2^, slice thickness = 3.4 mm, and number of volumes = 150. In the resting-state fMRI session, subjects were asked to lie on a bed and try to think about nothing about nothing with closed eyes.

### Data analysis

All data were analyzed with Brainvoyager QX software (Brain Innovation B.V., Maastricht, Netherlands). Structural images were spatially normalized through Talairach transformation with Brainvoyager QX. In the first part of the transformation, we detected the anterior and posterior commissure as landmarks for the y-axis of the Talairach coordinate system. In the anterior commissure, the x-axis runs from the left to the right hemisphere, while the z-axis runs from the inferior to the superior part of the brain. All boundaries were decided manually. After the anterior-posterior commissure transformation, the brain was separated by 12 sub-cuboids. These 12 sub-cuboids were expanded or shrunken linearly to correspond to the size standard Talairach brain sub-cuboids. These normalized T1 images were corrected by an intensity inhomogeneity correction (Sled et al., 1997; Hou et al., 2006). After this correction, non-cortical structures [other than GM, WM, and cerebral spinal fluid (CSF)] were removed by a brain extraction process. The extracted T1 images were segmented by several types of brain tissues (i.e., GM, WM, and CSF) with different ranges of intensities.

For cortical thickness measurements, we first defined boundary voxels, one at the WM–GM boundary and one at the GM–CSF boundary. The values of those boundary voxels were kept, and the intensities between the GM voxels were smoothed by Laplace's equation (Jones et al., 2000). From the smoothed values, the gradient value of each voxel was calculated and then streamlines with those values were made. To calculate cortical thickness, we checked the gradient and evaluated along the gradient direction. This process was repeated until we could approach other boundaries, such as WM or CSF. The sum of the process provides the cortical thickness. Cortical thickness measures were calculated by processes within Brainvoyager QX. Individual cortical thickness measures were aligned to each normalized cortex mesh for group analysis. Cortical thickness maps were calculated for all subjects.

Region of interest (ROI)-based analysis is known to be very useful in studies of specific brain areas (Gur et al., 2000; Singh et al., 2014; Kauttonen et al., 2015). Therefore, we performed a ROI-based analysis wherein the postcentral gyrus was defined as a ROI containing somatosensory areas (Biswal et al., 1995; Stippich et al., 1999; Ruben et al., 2001; Nguyen et al., 2004; Miyamoto et al., 2006). Cortical thickness maps of the two groups were analyzed by a two sample *t*-test in the ROI, and the result was corrected for multiple comparison at a cluster-level threshold of *p* < 0.005 by Brainvoyager QX (Forman et al., 1995; Goebel et al., 2006).

One subject in the non-music playing group was excluded from the analysis of resting-state functional images because of large head motion. The images of 25 subjects were preprocessed by slice scan time correction, 3D motion correction, and high pass temporal filtering (only signals with relatively high frequency >0.01 Hz). These functional images were smoothed with 6-mm FWHM and coregistered with each structural image. All resting-state functional images were analyzed by seed-based correlation analysis using Brainvoyager QX. In the analysis, the time course of seed ROIs were correlated with whole brain and correlation maps were created. Seed ROIs were those brain regions that showed different cortical thickness between the music and non-music playing groups. Individual correlation maps were made from seed ROIs. Correlation maps were analyzed by two sample *t*-test for two groups, and the resultant map was corrected for multiple comparison at a cluster-level threshold of *p* < 0.05.

## Results

### Cortical thickness analysis

**Figure 1:**
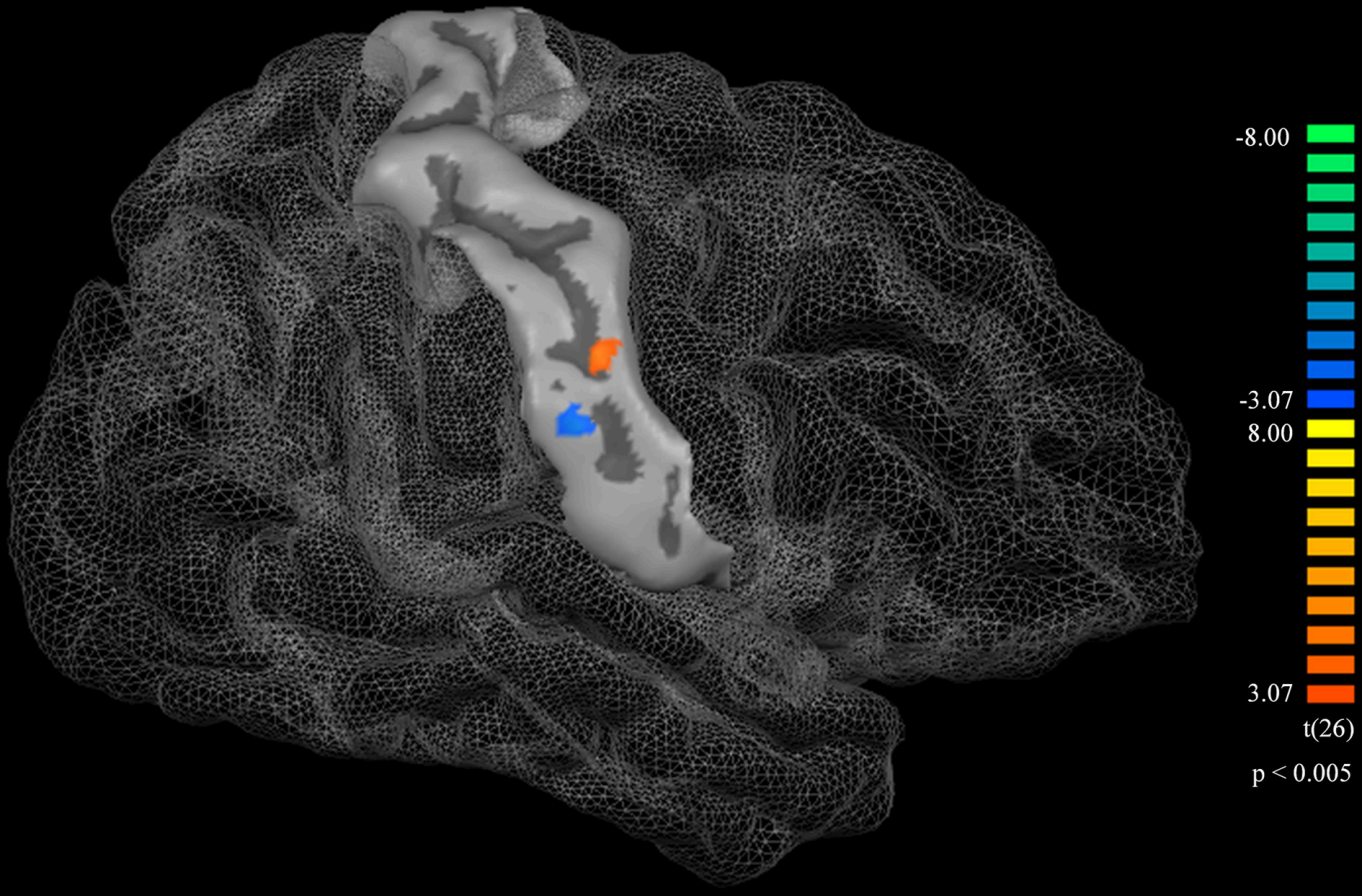
**A surface atlas showing of the postcentral gyrus and a cortical thickness difference map**. The orange represents the area in which the music playing group had greater cortical thickness than the non-music playing group. The blue represents the area in which the music playing group had less cortical thickness than the non-music playing group. Both thicker and thinner areas were statistically significant (*p* < 0.005, corrected).

**Figure 2:**
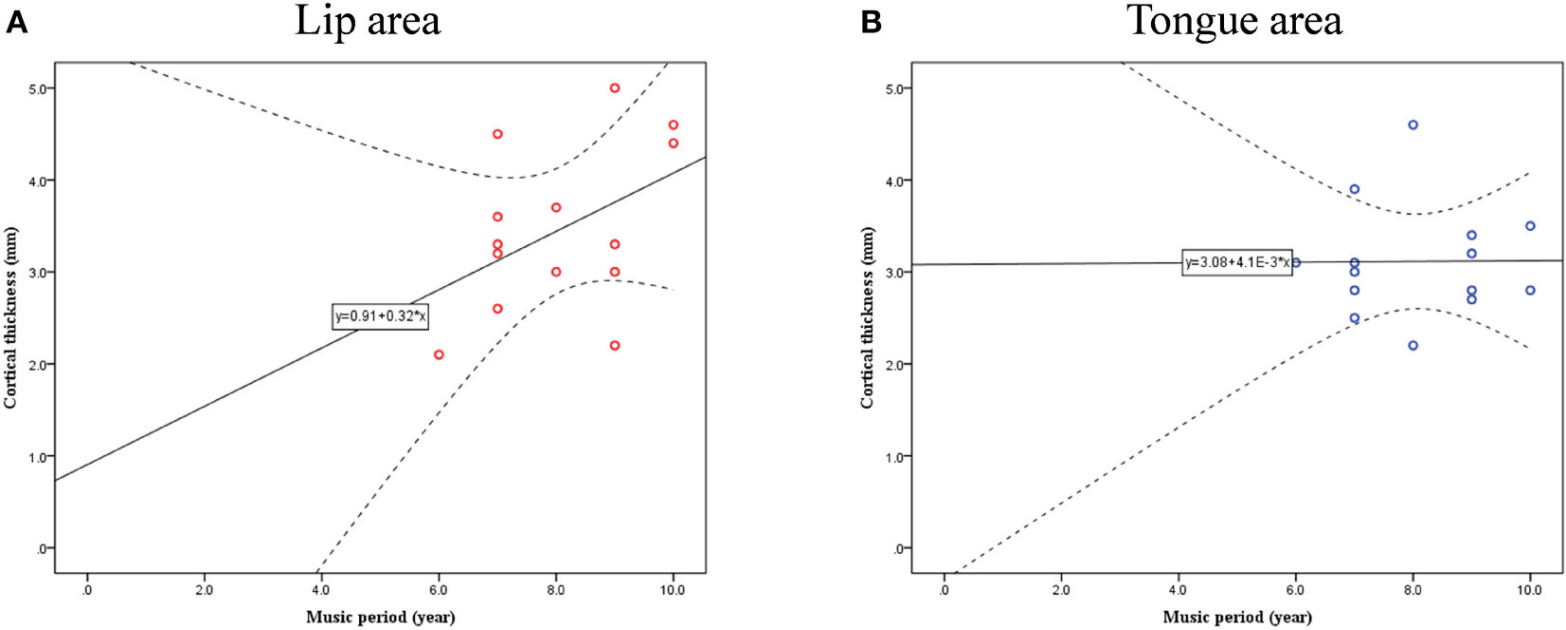
**Confidence interval (CI) for cortical thickness and musical training years in lip- (A) and tongue-related (B) brain areas**.

**Figure 3:**
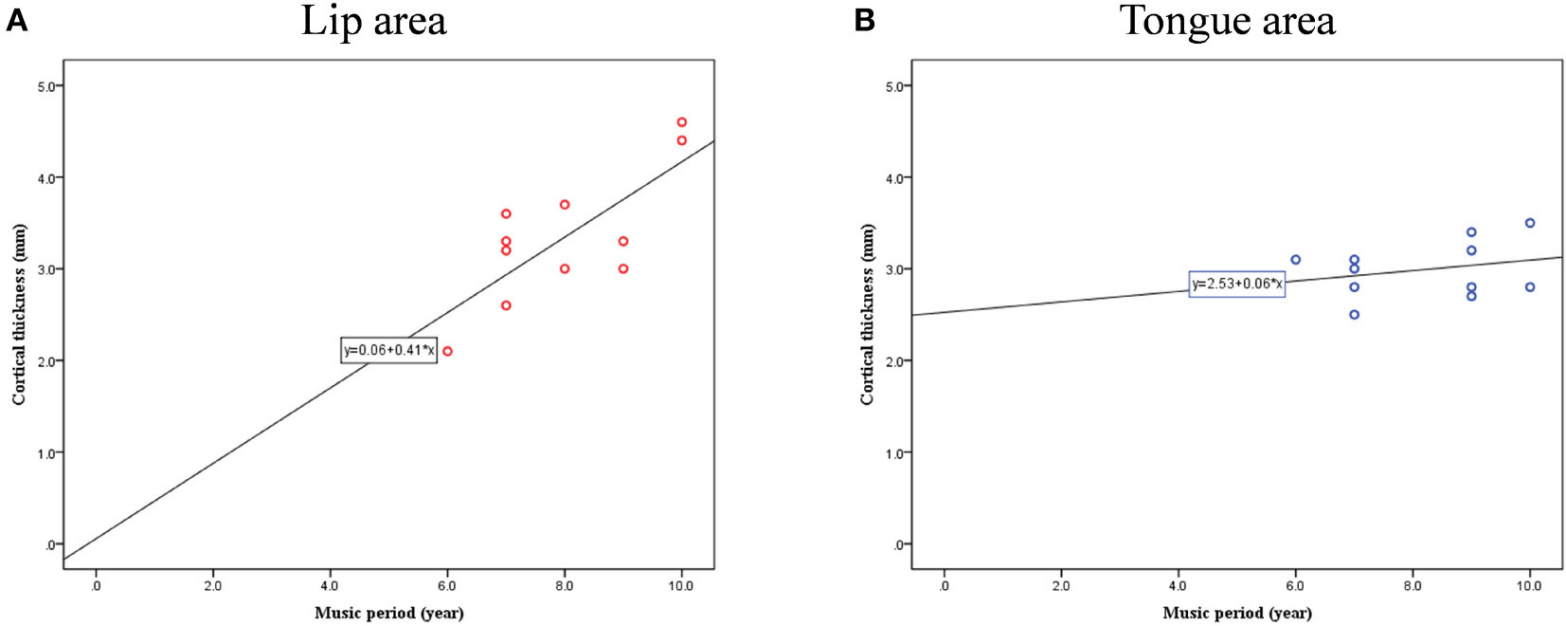
**Correlation between cortical thickness and musical training years in lip- (A) and tongue-related (B) brain areas**.

Cortical thickness difference maps for music and non-music playing groups were evaluated using a two sample *t*-test (*p* < 0.005, corrected). Two brain areas, which showed significant differences between the two groups, were found in the postcentral gyrus of the right hemisphere (Figure 1). The music playing group showed a thicker cortex in an area of the anterior part of the postcentral gyrus corresponding to the lips (Breshears et al., 2015) [*t*-value = 3.41; *p*-value = 0.003; cluster size = 9.71 mm^2^; Brodmann's area (BA) 3; Talairach coordinates: x = 58, y = −14, z = 44] but a thinner cortex in an area of the posterior part of the postcentral gyrus corresponding to the tongue (Breshears et al., 2015) [*t*-value = − 3.51; *p*-value = 0.002; cluster size = 10.93 mm^2^; BA2; Talairach coordinates: x = 64, y = −14, z = 35] compared with the non-music playing group. Areas with significantly different cortical thickness were subjected to ROI-based correlation analysis of thickness and years of musical training (association analysis). In the lip-related area of the postcentral gyrus, three participants of the music playing group were found as outliers at the 99% confidence interval (Figure 2A). Therefore, these three data points were excluded, and the remaining 11 subjects' data were used for the correlation analysis, which revealed significant and positive high correlations between the thickness and years of musical training (*r* = 0.761, *p* = 0.006 by Pearson correlation; Figure 3A). This means that changes in cortical thickness resulted from musical training. In the same way, in the tongue-related area, three participants in the music playing group were also found as outliers at 99% confidence interval (Figure 2B). Therefore, these three data points were excluded, and the remaining 11 subjects' data were used for the correlation analysis. However, there was no significant correlation between the thickness and years of musical training in this area (*r* = 0.262, *p* = 0.437 by Pearson correlation; Figure 3B).

### Resting-state fMRI analysis

**Figure 4:**
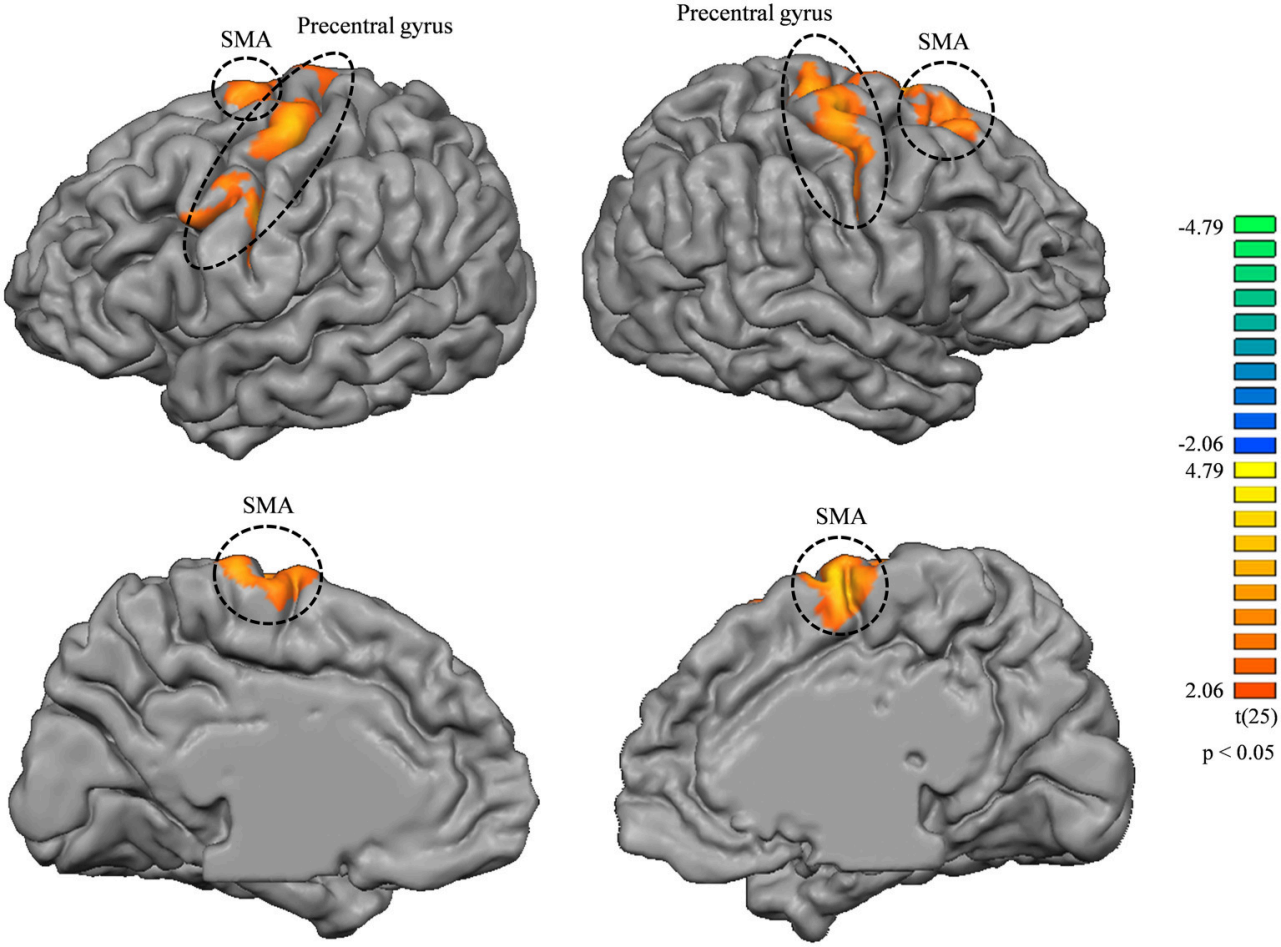
**Correlation map differences between music and non-music playing groups from lip-related brain areas (higher correlation in the music playing group); left column, left hemisphere; right column, right hemisphere**. Both brain hemispheres showed differences in the precentral gyrus and SMA bilaterally. All areas were corrected for multiple comparison at a cluster-level threshold of *p* < 0.05.

Seed-based correlation analysis of tongue-related areas of the brain did not show any significantly different maps between the music and non-music playing groups. Correlation analysis of lip-related areas revealed some significantly different maps between the two groups (higher correlation in the music playing group) (*p* < 0.05, corrected) in the precentral gyrus and supplementary motor areas (SMA) bilaterally (BA6; Figure 4). The correlation map located in the lower part of the precentral gyrus of the left hemisphere (BA6) was in the motor-related mouth region and corresponded to the somatosensory mouth region. Correlation maps located in the middle part of the precentral gyrus (BA4) of both hemispheres were in motor-related hand regions (the middle part was clearly separated from upper and lower parts in the left hemisphere but not separated from those in the right hemisphere). These results indicate that cortical thickness changes in somatosensory areas were related to new neuronal network developments in primary and supplemental motor areas.

## Discussion

The aim of our study was to determine how musical training with wind instruments causes structural and functional changes in the brain that could be attributed to specific ways of playing instruments. We mostly focused on somatosensory areas. Participants in this fMRI study were university students who received several years of training on wind instruments in junior and senior high school and are now members of reputable university wind ensembles. Participants chosen as non-music playing were university students with no special musical training. Cortical thickness analysis of structural plasticity in the brain showed significant differences in two areas of the postcentral gyrus associated with the lips and tongue according to previous reports (Pulvermüller et al., 2006; Meister et al., 2007; Brown et al., 2008; Venezia et al., 2012). In addition, correlation analysis in the lip-related area revealed a significant positive correlation between the thickness and years of musical training demonstrating that cortical thickness changes were longitudinal. In addition, seed-based correlation analysis by resting-state fMRI also revealed some areas that significantly differed between the music and non-music playing groups in the correlation map of lip-related areas, including those within the precentral gyrus and SMA, which are known to be related to learning mouth and motor skills and musical activity (Chen et al., 2012; Grabski et al., 2012; Gebel et al., 2013; Kim and Shin, 2014).

Along with changes in lip- and tongue-related brain areas, some reports suggest that playing wind instruments could result in physical changes to related body parts (i.e., the tongue and lips). For example, the muscles of the lips and tongue may change while playing a wind instrument (Methfessel, 1990). Also, lips are crucial to musicians who use only a single reed that physically produces sound on a wind instrument (Fuhrimann et al., 1987; Benade, 1990; Chen and Weinreich, 1996). Therefore, training with wind instruments can physically alter the lips and tongue, leading to changes in corresponding somatosensory brain areas (Draganski et al., 2006) as found in the current study. The positive correlation between the cortical thickness and training period in lip-related brain areas strongly suggests that changes in these areas were caused by musical training over several years.

The thickening of lip-associated brain areas can be explained in the same way as increased GM volume in certain areas associated with other types of training, such as juggling and piano playing. In contrast, the cause of cortical thinning in tongue-related brain areas is not easy to explain. Explanations for the cortical thickness decrease can be found in several previous studies. One explanation stems from previous studies showing sharpening in populations of neurons frequently recruited for a specific function (Desimone, 1996; Wiggs and Martin, 1998; Grill-Spector et al., 2006). Another possible explanation is that an increase in WM may cause a decrease in the ratio of GM in an imaging voxel as shown from some reports in which WM increased with musical training (Bengtsson et al., 2005; Zatorre, 2013). However, further study is needed to clarify the cause of this decrease as it relates to wind instrument training.

By resting-state fMRI, the music playing group showed stronger correlations with some areas in the precentral gyrus and SMA, and the relationship between precentral areas and SMAs with music has been found in previous studies. The precentral gyrus has been shown to be involved in musical instrument playing (Schieber, 1990; Amunts et al., 1997), musical training (Lin et al., 2002), and musical ability (Schlaug, 2001), while SMAs have been shown to be involved in musical training, especially in brain networks associated with rhythmic motor movements (Zatorre et al., 2007). In addition, the precentral gyrus and SMA are also known to interact closely for learning of musical instruments (Kim and Shin, 2014).

Unfortunately, the current study is limited in its design. Because the present study was designed to be cross-sectional, we cannot exclude the possibility that the music playing group members' abilities come from certain anatomical advantages over those of the non-music playing group rather than functional changes resulting from musical training. However, the positive correlation between the cortical thickness and years of musical training in lip-related brain areas suggests otherwise. This longitudinal evidence and the appearance of the new functional neuronal network in the music playing group support our interpretation that cortical thickness changes reflect changes in neuronal plasticity by musical training.

In conclusion, we found structural and functional changes in lip- and tongue-related areas in the postcentral gyrus related to longitudinal musical training with wind instruments. These results indicate that high-intensity musical training can change the structure and function of the brain. Furthermore, the current findings support the idea of altered neuroplasticity by musical training.

### Conflict of interest statement

The authors declare that the research was conducted in the absence of any commercial or financial relationships that could be construed as a potential conflict of interest.
